# A FTIR microspectroscopy study of the structural and biochemical perturbations induced by natively folded and aggregated transthyretin in HL-1 cardiomyocytes

**DOI:** 10.1038/s41598-018-30995-5

**Published:** 2018-08-21

**Authors:** Diletta Ami, Paolo Mereghetti, Manuela Leri, Sofia Giorgetti, Antonino Natalello, Silvia Maria Doglia, Massimo Stefani, Monica Bucciantini

**Affiliations:** 1 Department of Biotechnology and Biosciences, University of Milano-Bicocca, Piazza della Scienza 2, 20126 Milano, Italy; 2 Department of Neuroscience, Psychology, Area of Medicine and Health of the Child of the University of Florence, Viale Pieraccini, 6, 50139 Florence, Italy; 3 Department of Experimental and Clinical Biomedical Sciences, University of Florence, Viale Morgagni 50, 50134 Florence, Italy; 4 Department of Molecular Medicine, Unit of Biochemistry, University of Pavia, Viale Taramelli 3/B, 27100 Pavia, Italy; 5 Interuniversity Center for the Study of Neurodegenerative Diseases (CIMN), Florence, Italy

## Abstract

Protein misfolding and aggregation are associated with a number of human degenerative diseases. In spite of the enormous research efforts to develop effective strategies aimed at interfering with the pathogenic cascades induced by misfolded/aggregated peptides/proteins, the necessary detailed understanding of the molecular bases of amyloid formation and toxicity is still lacking. To this aim, approaches able to provide a global insight in amyloid-mediated physiological alterations are of importance. In this study, we exploited Fourier transform infrared microspectroscopy, supported by multivariate analysis, to investigate *in situ* the spectral changes occurring in cultured intact HL-1 cardiomyocytes exposed to wild type (WT) or mutant (L55P) transthyretin (TTR) in native, or amyloid conformation. The presence of extracellular deposits of amyloid aggregates of WT or L55P TTR, respectively, is a key hallmark of two pathological conditions, known as senile systemic amyloidosis and familial amyloid polyneuropathy. We found that the major effects, associated with modifications in lipid properties and in the cell metabolic/phosphorylation status, were observed when natively folded WT or L55P TTR was administered to the cells. The effects induced by aggregates of TTR were milder and in some cases displayed a different timing compared to those elicited by the natively folded protein.

## Introduction

The pathologic presence of extracellular or intracellular insoluble fibrillar deposits of well identified peptides/proteins in specific organs and tissues is a shared feature of amyloid diseases. The process by which natively folded peptides/proteins undergo ordered fibrillar aggregation appears to be multifactorial. It is different in various types of amyloidogenic molecules and depends on environmental conditions including pH, temperature, agitation, ionic strength, presence of surfaces or interacting molecules, and others^1^. A large number of amyloid diseases are neurodegenerative conditions since neurons, as post-mitotic cells, are particularly susceptible to the intracellular accumulation of damaged and misfolded proteins resulting from a disequilibrium in the mechanisms of proteostasis^2^. In addition to neurodegenerative diseases, protein misfolding is related to a number of systemic diseases, such as senile systemic amyloidosis (SSA), a condition that affects around 25% of the population over 80 years, and the rarer familial amyloid polyneuropathy (FAP), an autosomal-dominant lethal disease. SSA is characterized by a buildup of wild-type TTR in amyloid fibrils as extracellular deposits even in cardiac tissue; in some cases, SSA may also be genetically determined^3,4^. The familial form, FAP, is associated with point mutations in TTR, most of which are destabilizing and accelerate the deposition of amyloid fibrils mostly in peripheral nerves, but also in several organs including heart, kidneys and ocular vitreous^5^. Polyneuropathy and cardiomyopathy are predominant signs of most TTR amyloidoses, whose most severe cases can be treated only by liver, and, when needed, heart transplantation^5^. The pathological features of systemic amyloidosis can be traced back to the concurrent presence of amyloid deposits and circulating amyloidogenic precursors. It is likely that, locally in the interstitial space, amyloid fibrils can directly influence the oligomerization of amyloidogenic protein precursors, thus making the proteins cytotoxic just where the amyloid deposits are localized.

Despite enormous research efforts, a detailed understanding of the molecular basis of the mechanisms leading to protein/peptide misfolding and aggregation, aggregate targeting to specific organs/tissues/cell populations and cell dysfunction is still lacking. It is likely that the alterations of specific biochemical and/or signaling pathways induced by the aggregates are functionally interrelated to each other and to other dysfunctions, such as redox and ion homeostasis and inflammation. The complexity of the issue highlights the need for a systemic approach to model the pathogenesis of amyloid at a network level. Systemic approaches, including metabolomics and proteomics, hold promise for a global and deeper insight in amyloid-mediated physiological alterations. A better knowledge of amyloid biology would be a premise to develop novel strategies aimed at interfering with the multiple pathogenic cascades induced by misfolded/aggregated peptides/proteins.

In this study, we applied Fourier transform infrared (FTIR) microspectroscopy, supported by multivariate analysis, to investigate the spectral changes occurring in intact cells exposed to TTR in native or amyloid conformation. FTIR microspectroscopy is a non-invasive and label-free tool that requires a very limited amount of material and allows obtaining a biochemical fingerprint of the sample under investigation, providing information on the content and structure of its main biomolecules, as well as on their chemical modifications^6,7^. In the case of complex biological systems, such as intact cells, this spectroscopic approach provides, within a single measurement, information on the main biomolecules found in the sample, including lipids, proteins, nucleic acids, and carbohydrates^6,7^.

In particular, we investigated by FTIR microspectroscopy the biochemical modifications occurring in HL-1 cells exposed to wild type TTR (TTR-WT) or to a highly amyloidogenic variant (TTR-L55P) associated with aggressive forms of FAP, either as such or at varying aggregation times. Since the FTIR spectra of biological systems are very complex, resulting from the overlapping absorptions of the main biomolecules in the sample, to extract the significant and non-redundant information contained in the spectra we exploited an appropriate multivariate analysis, a tool allowing to process very large data sets. Accordingly, to reduce the dimensionality and to classify the spectra, we processed the FTIR data by the PCA-LDA tool (principal component analysis – linear discriminant analysis)^8^. Subsequently, a linear mixed model^9^ was built, using as variables the PCA-LDA scores; this approach was able to provide a statistical assessment taking into account the errors in the measurements. To assign biological significance to the FTIR data, the latter were compared with those resulting from biochemical experiments carried out on the same cells. Overall, our study aims at providing novel information on the amyloid toxicity theme by an innovative approach to describe a global cell signature resulting from exposure to natively folded or misfolded/aggregated TTR. To this purpose, different functional groups of macromolecules and their changes induced by externally added misfolded/aggregated TTR were monitored *in situ* in cultured intact HL-1 cardiomyocytes.

## Results

### Effects of TTR-WT and TTR-L55P in different conformational states on HL-1 cells: evaluation of ROS production and autophagy activation

**Figure 1:**
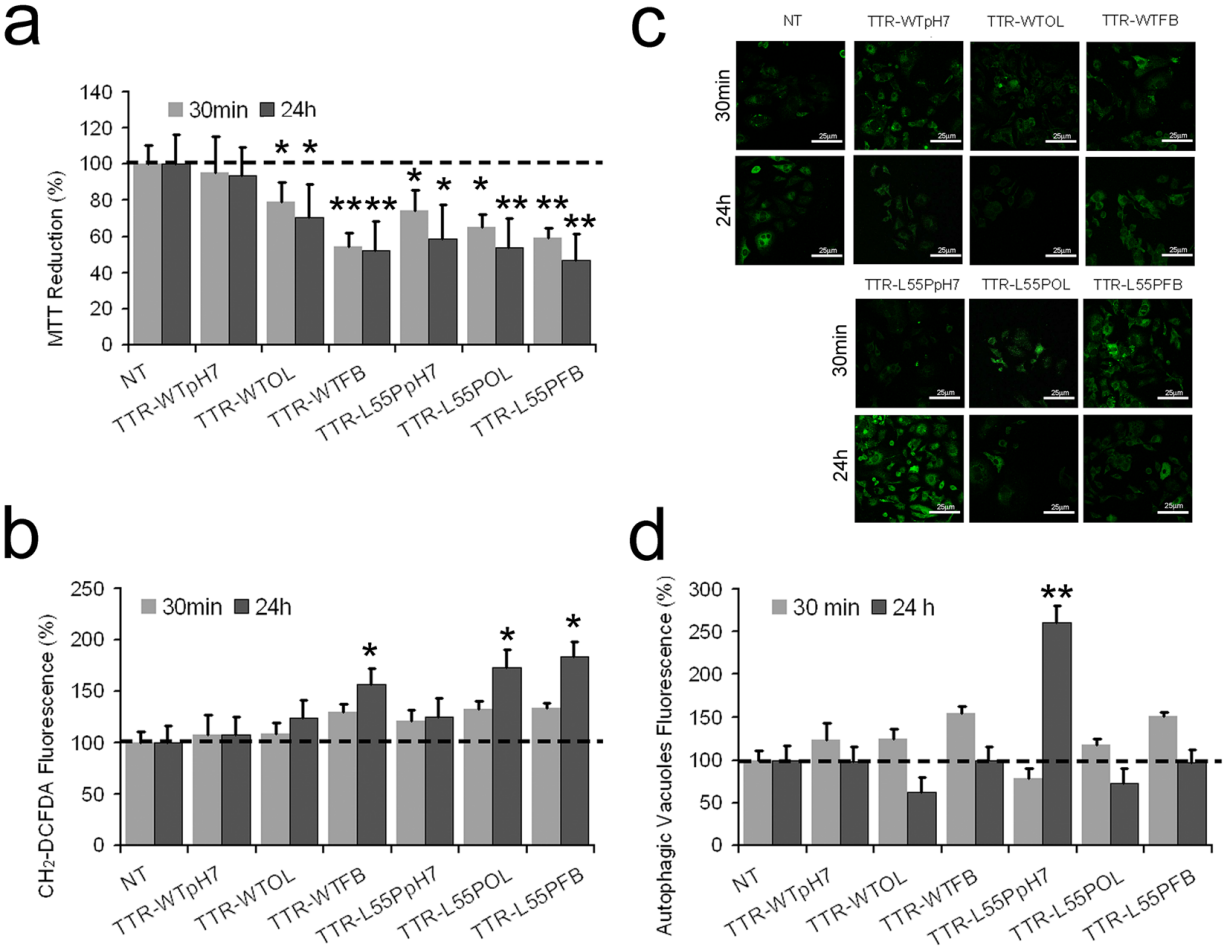
Cytotoxicity induced by misfolded TTR. HL-1 cells were untreated (NT) or treated for 30 min (gray) or 24 h (black) with 5.0 μM TTR-WT or TTR-L55P in native form (pH7), in oligomeric- (OL) or in the fibrillar-like (FB) amyloid conformation. (**a**) Cell viability. The viability of HL-1 cells was assessed by the MTT reduction assay. Data are mean ± SEM, n = 9. (**b**) ROS production. ROS production in HL-1 cells exposed for 30 min or 24 h to 5.0 μM TTR-WT or TTR-L55P in different conformations. Data are mean ± SEM, n = 9. (**c**,**d**) Autophagy detection. Representative confocal fluorescence images of autophagic vacuoles in HL-1 cells (**c**) and summarized data showing the number of autophagic vacuoles including pre-autophagosomes, autophagosomes, or autophagolysosomes by using the Cyto-ID^®^ probe. Data are mean ± SEM, n = 10–22 (d). Statistical analysis: One-way ANOVA-Dunnet’s test: *p < 0.05; **p < 0.01; vs control.

Initially, we analysed the viability and reactive oxygen species (ROS) production in HL-1 cardiomyocytes exposed for 30 min or 24 h to TTR-WT or to TTR-L55P in different conformational states: native (pH 7), oligomeric- (OL) or fibrillar-like (FB) aggregates, previously characterized (Figs 1, S1, S2)^10,11^. Figure 1a shows that, after 30 min and 24 h of exposure, the late FB aggregates of both TTR-WT and TTR-L55P were cytotoxic and induced a significant increase of ROS production. We observed toxicity also when the cells were incubated with TTR-WT or TTR-L55P in OL conformation and, although to a lesser extent, with the monomeric misfolded TTR-L55P kept at neutral pH. The FB species of both TTR-WT and TTR-L55P, as well as the OL species of TTR-L55P induced a significant increase of ROS in the exposed cells, in particular after 24 h of incubation (Fig. 1b).

Most often, cells undergoing oxidative stress activate an autophagic response aimed at clearing oxidized proteins by proteolysis^12^. Autophagy is an evolutionary conserved catabolic process that ensures continuous removal of damaged cell organelles and long-lived protein aggregates to maintain cellular homeostasis^13,14^. In the heart, autophagy is observed in a variety of human pathologies, where it can be either adaptive or maladaptive, depending on the context^15^. Therefore, we checked whether autophagy was induced in HL-1 cells following exposure to the different misfolded/amyloid species. Autophagy was monitored by confocal analysis using a dye that exhibits bright green fluorescence when selectively labels autophagic vacuoles, including pre-autophagosomes, autophagosomes, or autophagolysosomes. Representative images for Cyto-ID^®^ fluorescence in HL-1cells are reported in Fig. 1c. It can be seen that natively folded TTR-L55P significantly increased the Cyto-ID^®^ fluorescence intensity, suggesting increased autophagic vacuoles in 24 h-exposed HL-1 cells. The FB species of both TTR-WT and TTR-L55P also increased the Cyto-ID^®^ fluorescence in 30 min-treated cells, although to a lesser extent and with lower significativity. The number of autophagic vacuoles in Fig. 1d were summarized to quantitate autophagy.

### FTIR analysis of HL-1 cell modifications induced by TTR-WT in different conformational states

Next, we sought to relate the above reported cellular data to the changes in the cell macromolecular content and structure induced by TTR-WT; to this purpose, we studied the infrared response of HL-1 cells exposed to the protein in different conformational states (natively folded, early oligomeric and late fibrillar-like conformation). As a control, we investigated the infrared response of untreated cells (NT). In Fig. S3a we reported the measured IR absorption spectra of the intact HL-1 cells under the conditions reported above. To better assign the selected IR bands to specific biomolecules, we analysed the second derivatives of the FTIR spectra, which allow resolving the different overlapping components in the complex absorption bands^16^. Furthermore, to assess the reproducibility and to identify the more significant spectral variations occurring in treated cells, we supported the FTIR data with a multivariate analysis approach, namely the principal component-linear discriminant analysis (PCA-LDA). Then, we applied a linear mixed model analysis to investigate the statistical significance of the differences detected in cells treated with the protein in the different conformational states and at different incubation times^8,9^. To this aim, a single parameter, η_G′,T_, was calculated to quantify the differences between the spectra of the treated cells compared to the spectra of the untreated sample (see Methods for details). Notably, the larger is the η_G′,T_ value, the more significant is cell perturbation induced by the different treatments.

### FTIR analysis of protein secondary structure modifications in HL-1 cells exposed to TTR-WT

**Figure 2:**
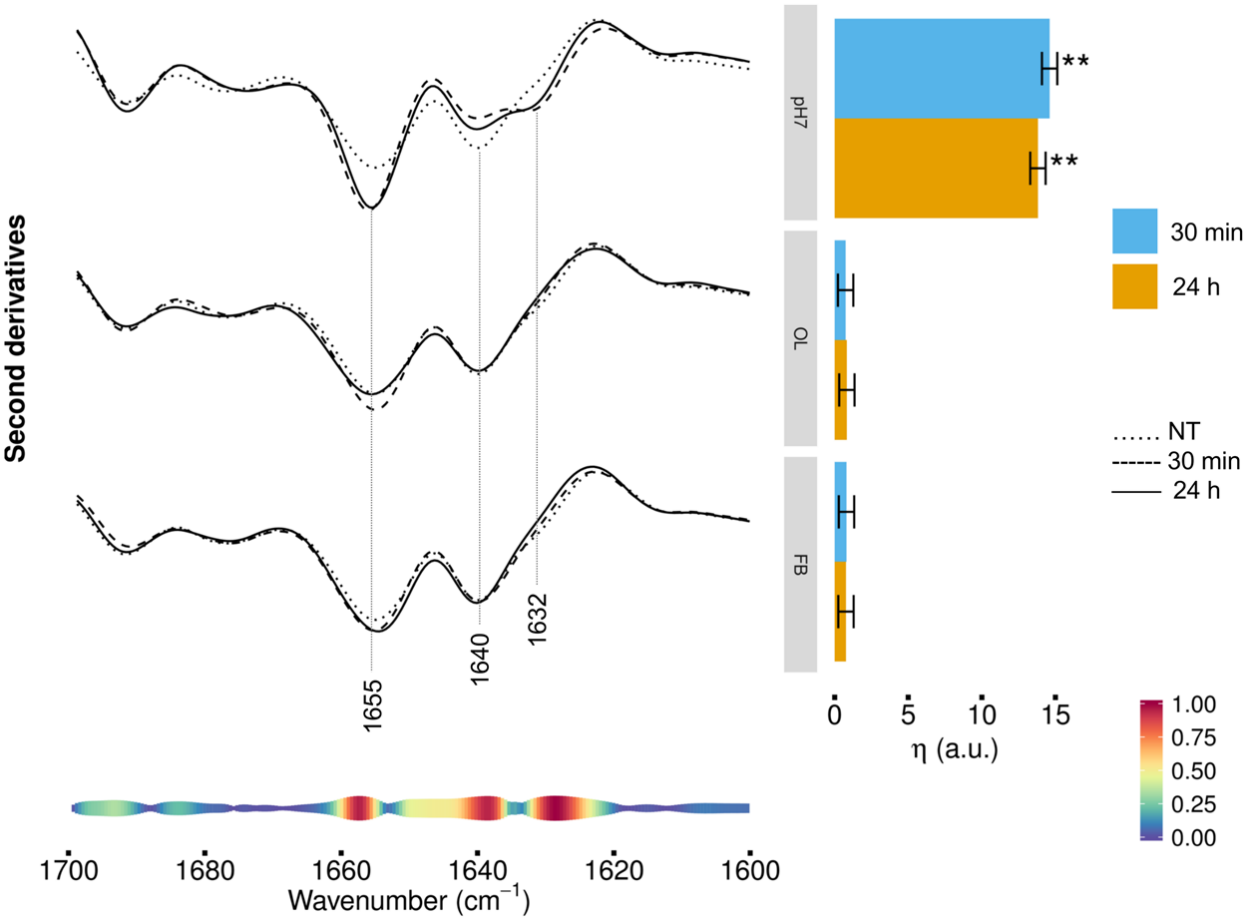
HL-1 cells incubated with TTR-WT: Amide I band analysis. Left panel: second derivatives of the FTIR absorption spectra of HL-1 cells untreated (dotted line), incubated for 30 min (dashed line) or 24 h (continuous line line) with TTR-WT in native conformation (top spectra), in the early oligomeric (middle spectra) or in the late fibrillar-like (bottom spectra) form. Second derivatives are shown after spectra normalization at Amide I band area. Representative spectra are shown, computed as average of the three most central spectra within the space formed by the first three PCA-LDA scores. At the bottom, the pseudoloadings are shown as coloured bands: larger, red-coloured bands indicate important wavenumbers, while smaller, blue-coloured bands indicate unimportant wavenumbers (see bottom-right colour bar). Representative wavenumbers are indicated on the plot. Right panel: differences of the average (across spectra) linear mixed model response variable (η, see Methods for details) between TTR-WT treated (pH7, OL, FB at 30 min and 24 h) and control (NT) cells. Error bars show the 95% confidence interval. Stars above the vertical bar indicate the Dunnet’s adjusted two-sided P-values from two-sample Student t-test. P-value: ** < 0.01.

Figure 2 (left panel) reports the second derivative analysis of the Amide I band, in the 1700−1600 cm^−1^ range, arising from the C=O stretching vibration of the peptide bond, which can give information on the whole secondary structures in cell proteins^17–19^. The control cell spectrum (NT) was characterized by a component at ~1655 cm^−1^, which arises from α-helix and random coil structures^17,19^. A large absorption that can be assigned to intramolecular β-sheets was detected at ~1640 cm^−1^, with a shoulder at low frequencies that might indicate the presence of intermolecular β-sheets typical of protein aggregates^18,19^. Cell incubation for 30 min with TTR-WT at pH7.0 (Fig. 2, left panel, top spectra) led to an increase in the intensity of the α-helix/random coil band, as well as to a reduction of the intramolecular β-sheet component at ~1640 cm^−1^. Moreover, a well resolved component at ~1632 cm^−1^, mainly assigned to intermolecular β-sheets^18,20^, appeared. Very similar spectral features were observed after 24 h of treatment. Then, cell treatment with oligomeric-like form of TTR-WT (Fig. 2, left panel, middle spectra) led to an increase of the intensity of the α-helix and random coil band at ~1655 cm^−1^, after 30 min of treatment, and to a slight decrease of the 1632 cm^−1^ shoulder mainly due to intermolecular β-sheets, detected at the longer time of incubation.

When cells were incubated with TTR-WT in the fibrillar-like conformation (Fig. 2, left panel, bottom spectra), starting from 30 min a weak increase of the α-helix and random coil band, and a slight decrease of the intermolecular β-sheets were observed.

These results were confirmed by the multivariate analysis, shown in Fig. 2 (right panel), showing the differences between cells treated with TTR-WT in the different conformational states and control cells. The differences are quantified by a single, η_G′,T_ value which measures the average separation between treated and control cells on the first PCA-LDA component (see Methods for details). The larger is η_G′,T_, the more dissimilar are the spectra of the challenged cells compared to untreated cells. Focusing on the Amide I band, HL-1 cells resulted significantly perturbed when incubated with native TTR-WT, while the early and the late aggregates had a minor impact.

On average, small differences between the two incubation times were observed, with a slightly greater effect for the shorter time (30 min). Furthermore, at the bottom of Fig. 2 (left panel) we reported the pseudoloading plots that, pinpointing the regions mostly contributing to spectra discrimination, led to identify particularly important the 1632–1625 cm^−1^, 1641–1638 cm^−1^, and 1659–1656 cm^−1^ spectral ranges. Therefore, in agreement with direct spectra inspection, these results indicate that the incubation with TTR, mostly when administered in native form, induced a rearrangement of the whole content of cell protein β-sheets, which became enriched in intermolecular structures, accompanied by an increase of the α-helix/random coil content.

The analysis has been extended to the 3600–3050 cm^−1^ spectral range, where the Amide A absorption, mainly due to the peptide bond NH stretching^19^, does occur (Fig. S4). In agreement with the above results, also in this case more intense spectral perturbations were observed when the cells were incubated with the protein in the natively folded form.

### FTIR analysis of HL-1 cells exposed to TTR-WT in the 1500-900 cm^−1^ spectral range

To better investigate the spectral modifications arising from cell incubation with TTR-WT, we explored the infrared response between 1500–900 cm^−1^, a very complex spectral range where lipid, carbohydrate, nucleic acid and protein absorption overlaps^7,19,21–24^. For sake of clarity, the FTIR characterization coupled to the multivariate analysis in the two selected subranges 1500–1350 cm^−1^ and 1200–900 cm^−1^ was reported separately, whereas the 1350–1200 cm^−1^ range was excluded due to the absence of significant differences.

**Figure 3:**
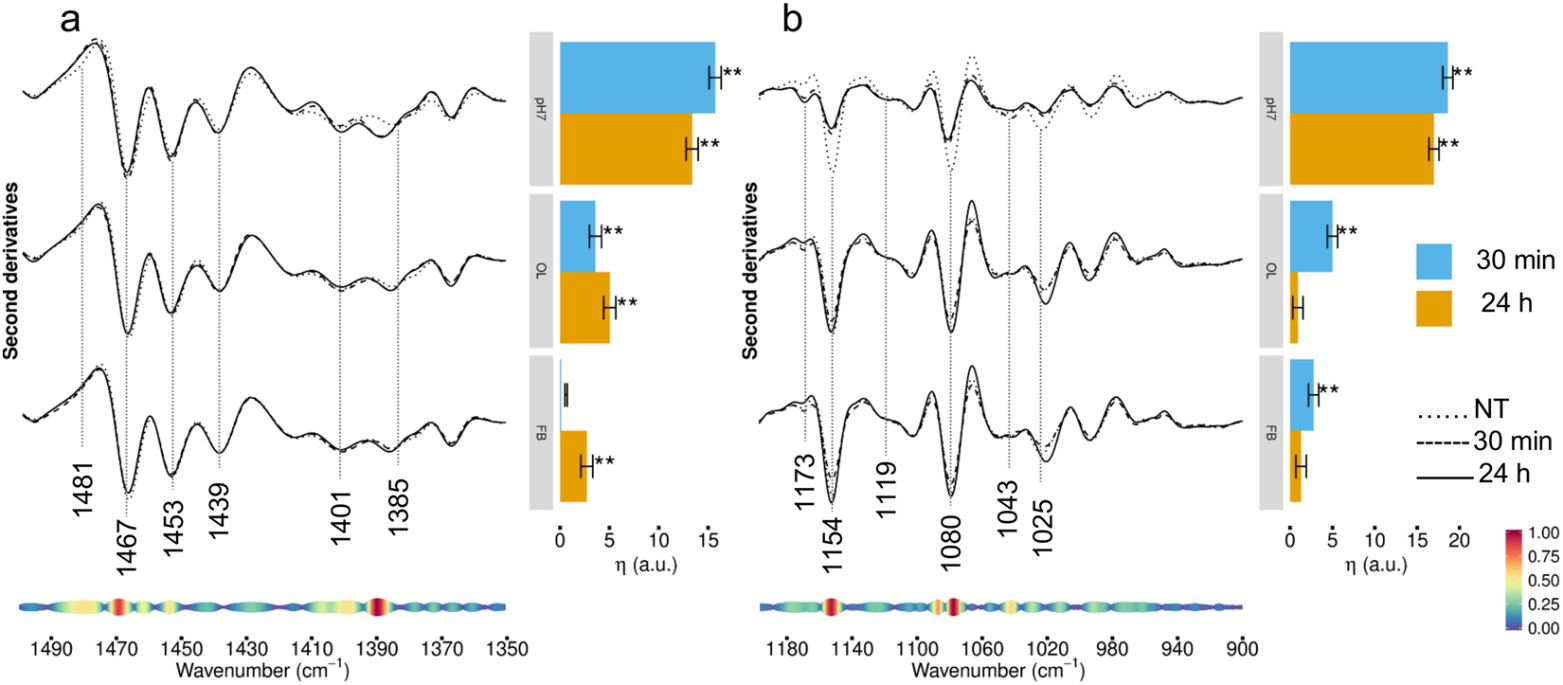
HL-1 cells incubated with TTR-WT: analysis of the 1500–900 cm^−1^ spectral range. Left panel: second derivatives of the FTIR absorption spectra of HL-1 cells untreated, incubated for 30 min or for 24 h with TTR-WT in native (top spectra), oligomeric- (middle spectra) and fibrillar-like (bottom spectra) conformation. The spectra are shown after normalization at Amide I band area, in two spectral ranges: 1500–1350 cm^−1^, mainly due to lipid absorption (panel a), and 1200–900 cm^−1^ (panel b). At the bottom, the pseudoloadings are shown as coloured bands: wider, red-coloured bands indicate important wavenumbers, while thinner, blue-coloured bands indicate unimportant wavenumbers (see bottom-right colour bar). Representative wavenumbers are indicated on the plot. Right panels: differences of the average (across spectra) linear mixed model response variable (η, see Methods for details) between TTR-WT treated (pH7, OL, FB at 30 min and 24 h) and control (NT) cells. Error bars show the 95% confidence interval. Stars above the vertical bar indicate the Dunnet’s adjusted two-sided P-values from two-sample Student t-test. P-value: ** < 0.01.

In Fig. 3a (left panel, top spectra), the second derivative spectra of cells treated with natively folded TTR-WT are compared with that of untreated cells in the 1500−1350 cm^−1^ spectral range, dominated by the absorption of lipid hydrocarbon chains and head groups. The control cell spectrum was characterized by three well resolved components - mainly due to lipid hydrocarbon chain deformation modes - respectively at ~1467 cm^−1^ (hydrocarbon chain CH_2_ bending and/or CH_3_ deformation), ~1453 cm^−1^ and 1439 cm^−1^ (CH_3_ deformation)^21,25,26^. A broad absorption around 1481 cm^−1^ was also observed, which could be partly assigned to N(CH_3_)_3_ group, typical of choline (antisymmetric bending) and to deformation vibrations of CH_2_ and CH_3_ groups from steroids, likely cholesterol^17,27^. Moreover, we detected two absorptions at ~1401 cm^−1^ and at ~1385 cm^−1^, respectively. While the first can be mainly assigned to the methyls of the choline N(CH_3_)_3_ group (symmetric bending)^17,21^, the latter arises from the CH_3_ of either the lipid hydrocarbon chain, and the protein amino acid side chains^28^. Interestingly, this component was also assigned to glycosylated proteins^29^.

At lower frequencies (Fig. 3b, left panel), the spectrum was dominated by the absorption of phosphate groups mainly from phospholipids and nucleic acids, as well as by the absorption of the C-O group in carbohydrates, including those bound to proteins and lipids^21–23^. Notably, the untreated cell spectrum displayed three well resolved and complex components at ~1154 cm^−1^, 1080 cm^−1^, 1025 cm^−1^, and a wide absorption at ~1043 cm^−1^, whose simultaneous presence might be indicative of glycogen absorption^30,31^. The 1080 cm^−1^ band is also due to the absorption of phosphates, thus including contributions from nucleic acids and phospholipids^21,23^, while the 1154 cm^−1^ band is due to the C-O groups of carbohydrates, as well as to the C-O stretching mode of the C-OH groups of serine, threonine and tyrosine^32^. Finally, the low intensity band detected at 1173 cm^−1^ is also assigned to non-hydrogen bonded C-O bond in the C-OH group of serine, threonine, and tyrosine^32^. Interestingly, the relative intensities of these two components could provide information on protein phosphorylation^32^.

Cell treatment with native TTR-WT resulted in significant spectral changes already at 30 min; in particular, we detected the upshift to 1389 cm^−1^ of the 1385 cm^−1^ band, which can tentatively be assigned to a decrease of protein glycosylation (Fig. 3a). In addition, since the 1389 cm^−1^ band is also assigned to lipid moieties^27^^,^^33^, the observed variation could also reflect a modification of lipid interactions with the surrounding molecules of the cell membranes, thus suggesting a variation of membrane lipid properties induced by TTR interaction/internalization.

At lower frequencies, we observed the simultaneous reduction in intensity of the three components at 1154 cm^−1^, 1080 cm^−1^, and 1025 cm^−1^, suggesting a reduction of the glycogen content. Noteworthy, the variation of carbohydrate absorption could support the hypothesis of a decrease of glycosylation discussed above. Moreover, the decrease of the 1154 cm^−1^ band intensity, also due to hydrogen-bonded C-O groups of serine, threonine, and tyrosine, occurred simultaneously to a slight increase of the 1173 cm^−1^ (non-hydrogen-bonded C-O of the C-OH groups). These results could indicate a partial loss of hydrogen bonding of the C-OH groups of serine, threonine and tyrosine residues, which might be due to a phosphorylation event likely induced by the incubation with TTR. Notably, the new minor absorption at 1119 cm^−1^ that appeared starting from 30 min of incubation, assigned to inorganic phosphate, is typical of phosphorylated enzymes^34^.

As confirmed by the multivariate analysis reported in the right panels of Fig. 3a and b, at 24 h incubation the effects induced by cell treatment with TTR-WT at pH7.0 were slightly reduced, yet comparable to those detected at the shorter incubation time. When the cells were treated with TTR-WT OL (Fig. 3a, middle spectra) or FB (Fig. 3b, bottom spectra) form, only modest effects, as compared to cell treatment with the natively folded protein (pH7.0), were observed. In particular, while the spectral variations affecting in particular the lipid moieties were more significant at 24 h incubation, those observed in the complex 1200-900 cm^−1^ range, reflecting an interplay of glycogen content and protein phosphorylation modifications, were more pronounced at the shorter incubation time in cells incubated with TTR-WT, both oligomeric- and fibrillar-like conformation.

The analysis of these complex spectral ranges took particularly advantage from the multivariate analysis, which enabled us to identify the most significant components responsible for the observed variance. As indicated by the pseudoloading plots at the bottom of Fig. 3a and b, these components were in particular the 1471–1467 cm^−1^ range, assigned to hydrocarbon chains CH_2_/CH_3_; the 1392-1388 cm^−1^ range, assigned to protein side chains as well as to lipid moieties; the 1481–1477 cm^−1^ range, partly assigned to choline N(CH_3_)_3_ group and to cholesterol CH_2_/CH_3_ groups, and the 1454–1453 cm^−1^ range, assigned to hydrocarbon tail CH_3_. Moreover, at the lower frequencies, the resulting most significant components were the 1156–1149 cm^−1^ and the complex absorption between 1089 and 1074 cm^−1^. Overall, these results indicate a possible involvement of cell glycogen, together with a modification of cell protein phosphorylation and glycosylation in response to cell incubation with natively folded TTR-WT.

### FTIR analysis of hydrocarbon chain modifications in HL-1 cells exposed to TTR-WT

**Figure 4:**
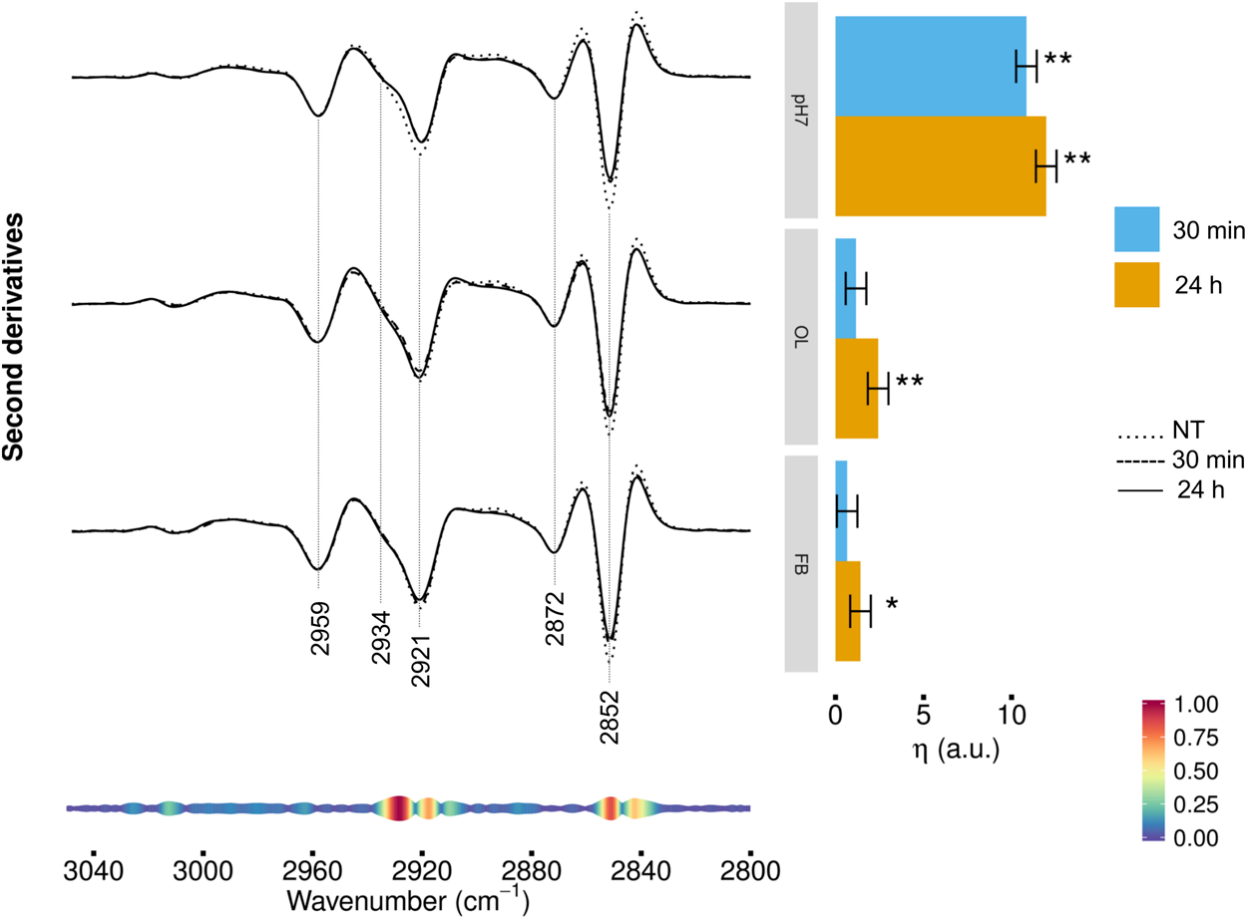
HL-1 cells incubated with TTR-WT: analysis of the 3050–2800 cm^−1^ CHn stretching range. Left panel: second derivatives of the FTIR absorption spectra of HL-1 cells untreated, incubated for 30 min or 24 h with natively folded TTR-WT (top spectra), OL- (middle spectra) or FB- (bottom spectra) like form. Second derivatives are shown after spectra normalization at the CH_3_ band at 2959 cm^−1^. Representative spectra are shown, computed as average of the three most central spectra within the space formed by the first three PCA-LDA scores. Right panel: differences of the average (across spectra) linear mixed model response variable (η, see Methods for details) between TTR-WT-treated and control (NT) cells. Error bars show the 95% confidence interval. Stars above the vertical bar indicate the Dunnet’s adjusted two-sided P-values from two-sample Student t-test. P-value: * < 0.05, ** < 0.01.

To investigate more in detail the effects of cell treatment with TTR-WT on cell lipids, we extended the infrared analysis to the 3050–2800 cm^−1^ spectral range, dominated by the absorption of lipid hydrocarbon chains. As shown in Fig. 4 (left panel), the spectrum of control cells was characterized by four main absorption bands due to the CH_2_ (at ~2921, 2852 cm^−1^) and CH_3_ (at ~2959 and 2872 cm^−1^) stretching vibrations of the lipid hydrocarbon chains^21,25^. We also detected a shoulder around 2934 cm^−1^, mainly assigned to CH_2_ vibrations that characterize the spectrum of cholesterol^35–37^. After 30 min of cell treatment with natively folded TTR-WT (Fig. 4, top spectra), some of these spectral features were modified; in particular, the CH_2_ bands at ~2921 cm^−1^ (antisymmetric stretching) and 2852 cm^−1^ (symmetric stretching) decreased in intensity, suggesting a rearrangement of the lipid hydrocarbon chain length that could affect membrane fluidity. Modest changes were found at both times of incubation when the cells were treated with TTR-WT OL and FB forms, as compared to those observed in cells exposed for the same times to the natively folded protein (Fig. 4, middle and bottom spectra). In agreement with the direct observation of the second derivative spectra, the multivariate analysis (Fig. 4, right panel) revealed a remarkable spectral variation in cells treated for both 30 min and 24 h with native TTR. The same response was modest in cells incubated for 30 min with early or late TTR-WT aggregates, while it became just more significant in 24 h-exposed cells.

As reported at the bottom of Fig. 4 (left panel), two spectral components were particularly important to determine the spectral variations in cells treated with TTR in the different conformational states: the component at 2932–2925 cm^−1^, mainly ascribable to cholesterol, but likely involving also the absorption of the CH_2_ groups in the hydrocarbon chains, and the component at 2853–2851 cm^−1^, assigned to the hydrocarbon chain CH_2_. Overall, in accordance with the analysis of the 1500–1350 cm^−1^ spectral range, these results reflect a minor, yet significant, rearrangement of membrane lipids in terms of hydrocarbon chain length and cholesterol content, both - particularly cholesterol - affecting bilayer fluidity^38^. In this perspective, we may speculate that cells challenged with TTR - particularly when administered in the natively folded conformation - rearrange their membranes as a part of the physiological response to natively folded TTR^39^.

### FTIR analysis of HL-1 cell modifications induced by TTR-L55P in different conformational states

Once investigated in detail the modifications of the FTIR spectral components in HL-1 cells exposed to TTR-WT in different conformational states, we extended our analysis to the effects induced by cell incubation with the pathogenic, particularly aggressive, TTR-L55P mutant, under the same experimental conditions. Similarly to the analysis reported for TTR-WT, we studied the infrared response (see Fig. S3b) of the cells challenged for 30 min or 24 h with the TTR-L55P, in the natively folded (pH7), oligomeric-(OL) or fibrillar-like (FB) form.

### FTIR analysis of protein secondary structure modifications in HL-1 cells exposed to TTR-L55P

**Figure 5:**
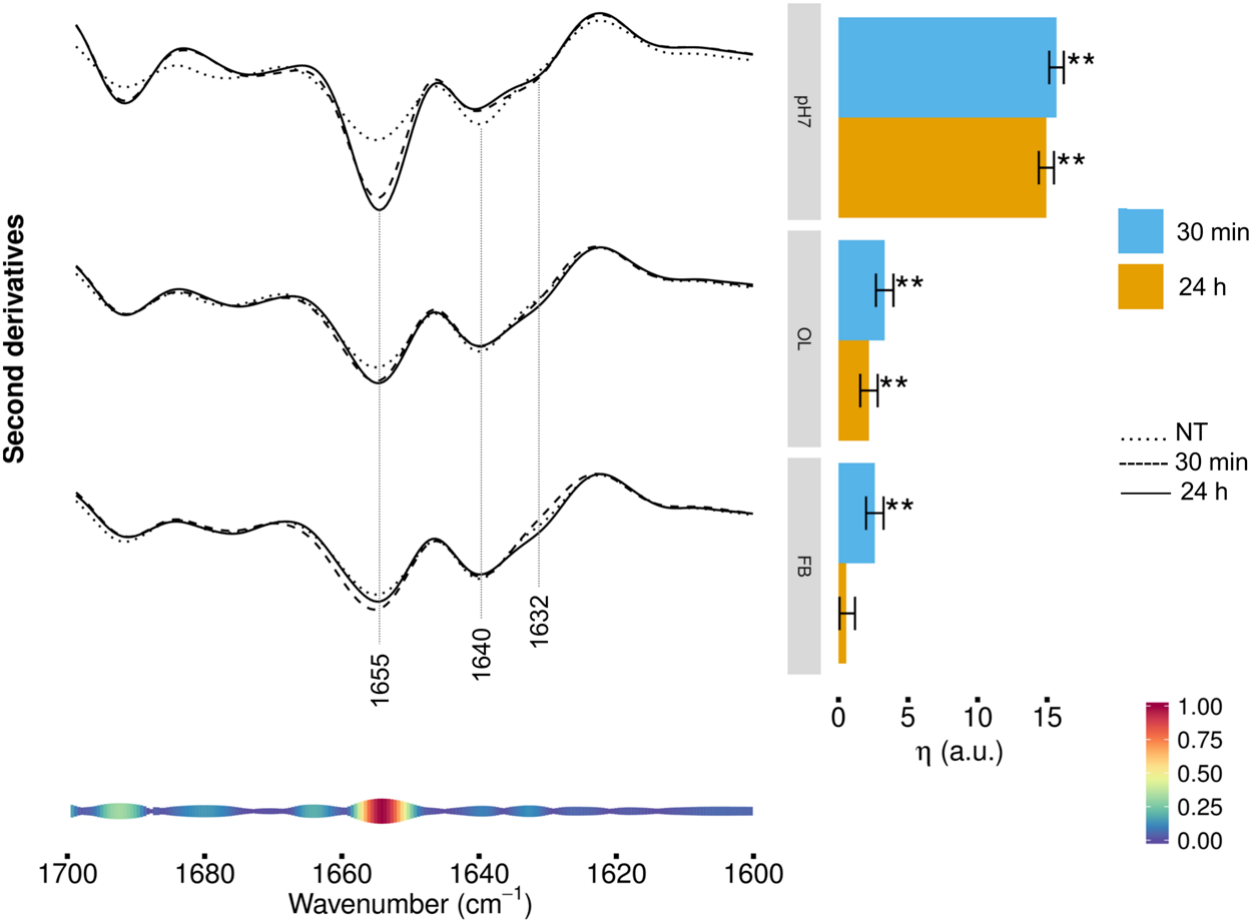
HL-1 cells incubated with TTR-L55P: Amide I band analysis. Left panel: second derivatives of the FTIR absorption spectra of HL-1 cells untreated (dotted line), incubated for 30 min (dashed line) or 24 h (continuous line) with TTR-L55P natively folded (pH7) (top spectra), in early oligomeric-like (OL) (middle spectra) or late fibrillar-like (FB) (bottom spectra) conformation. Second derivatives are shown after spectra normalization at Amide I band area. Representative spectra are shown, computed as average of the three most central spectra within the space formed by the first three PCA-LDA scores. At the bottom, the pseudoloadings are shown as coloured bands: wider, red-coloured bands indicate important wavenumbers, while thinner, blue-coloured bands indicate unimportant wavenumbers (see bottom-right colour bar). Representative wavenumbers are indicated on the plot. Right panel: differences of the average (across spectra) linear mixed model response variable (η, see Methods for details) between TTR-L55P-treated (pH7, OL, FB at 30 min and 24 h) and control (NT) cells. Error bars show the 95% confidence interval. Stars above the vertical bar indicate the Dunnet’s adjusted two-sided P-values from two-sample Student t-test. P-value: ** < 0.01.

As observed for TTR-WT, the TTR-L55P variant also induced a few spectral modifications in the 1700–1600 cm^−1^ range, particularly in cells exposed to the protein in the natively folded conformation at pH7.0 (Fig. 5, left panel, top spectra). Notably, we detected a significant increase in the intensity of the α-helix/random coil component at ~1655 cm^−1 ^^17,19^ as compared to untreated cells. The effect of cell treatment with the protein was evident already at 30 min of incubation and continued throughout the observation time. We also observed a slight decrease of the intensity of the native β-sheet component at 1640 cm^−1^, which was simultaneous to a minor increase of the shoulder at 1632 cm^−1^, mainly due to the intermolecular β-sheets typical of protein aggregates^18,19^. The pseudoloading plot (bottom of Fig. 5, left panel**)** identified the spectral component in the 1657–1652 cm^−1^ range as an important signature of the spectral difference among the different treatments, suggesting that cell incubation with TTR-L55P affected mainly the α-helix/random coil structures of cell proteins.

In agreement with the multivariate analysis reported in Fig. 5 (right panel), cell treatment with TTR-L55P in the oligomeric- (Fig. 5, left panel, middle spectra) or fibrillar-like (Fig. 5, left panel, bottom spectra) conformation induced minor changes in the Amide I spectral features, as compared to the natively folded protein (pH7). This suggests that the overall secondary structures of cell proteins were not remarkably affected by cell incubation with the protein in these conformational states even though, in all cases, the observed spectral variations were just more pronounced at the shorter incubation time.

As discussed for the cells treated with TTR-WT, also in this case the Amide A band analysis (Fig. S4) confirmed a higher cell perturbation upon cell treatment with the protein in its natively folded conformation.

### FTIR analysis in the 1500–900 cm^−1^ spectral range of HL-1 cells exposed to TTR-L55P

**Figure 6:**
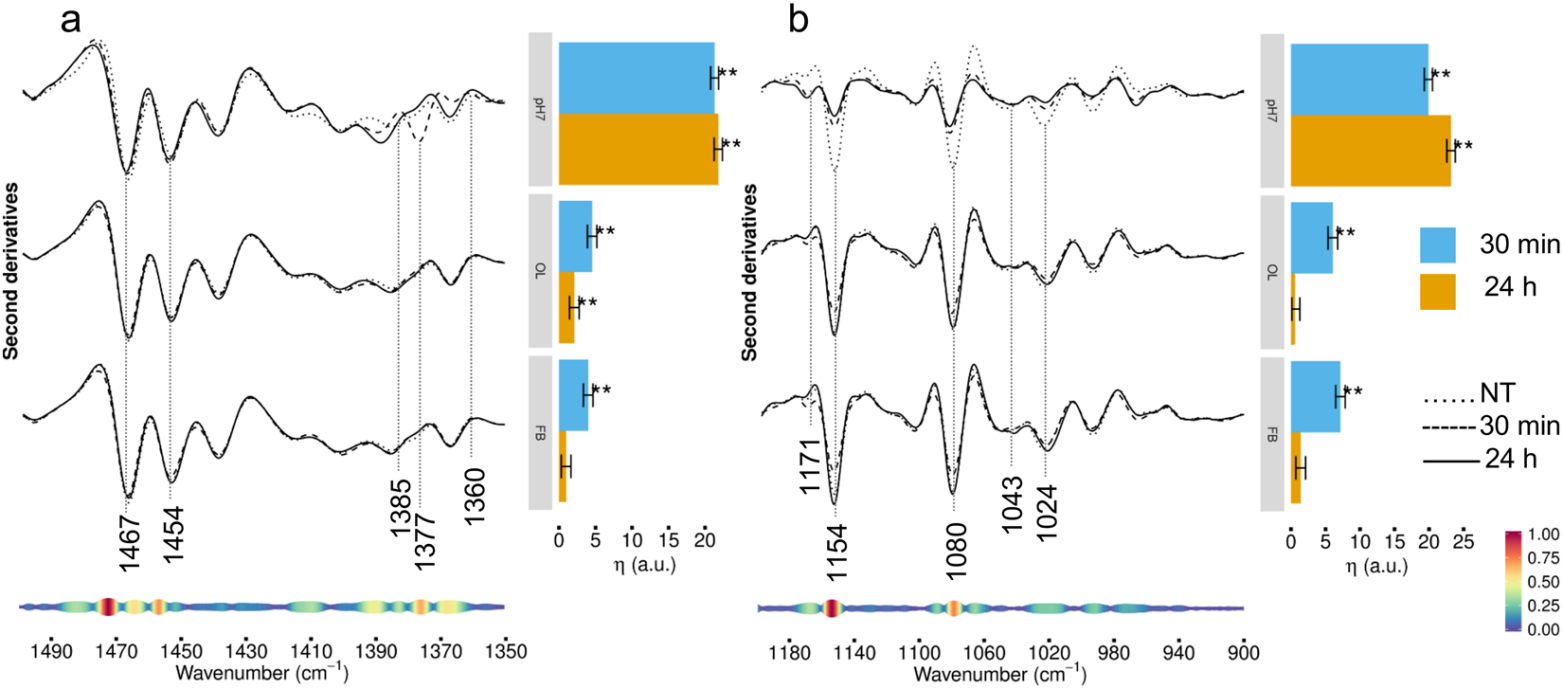
HL-1 cells incubated with TTR-L55P: analysis of the 1500–900 cm^−1^ spectral range. Left panel: second derivatives of the FTIR absorption spectra of HL-1 cells untreated, incubated for 30 min or 24 h with TTR-L55P in the natively folded (top spectra), oligomeric- (middle spectra) or fibrillar-like (bottom spectra) conformation. Second derivatives are shown after spectra normalization at Amide I band area, in two spectral ranges: 1500-1350 cm^−1^, mainly due to lipid absorption (panel a), and 1200-900 cm^−1^ (panel b). At the bottom, the pseudoloadings are shown as coloured bands: wider, red-coloured bands indicate important wavenumbers, while thinner, blue-coloured bands indicate unimportant wavenumbers (see bottom-right colour bar). Representative wavenumbers are indicated on the plot. Right panels: differences of the average (across spectra) linear mixed model response variable (η, see Methods for details) between TTR-L55P-treated (pH7, OL, FB at 30 min and 24 h) and control (NT) cells. Error bars show the 95% confidence interval. Stars above the vertical bar indicate the Dunnet’s adjusted two-sided P-values from two-sample Student t-test. P-value: ** < 0.01.

In Fig. 6a (left panel) we compared the second derivative spectra in the 1500–1350 cm^−1^ range of cells exposed for 30 min or 24 h to natively folded TTR-L55P (pH7.0) (top spectra), with that of untreated control cells. In particular, starting from 30 min of incubation we detected a variation of the absorption at ~1467 cm^−1^, mainly assigned to CH_2_ and/or CH_3_ groups of hydrocarbon chains^21,25,26,40^. Considering that this component is sensitive to lipid phase transition/organization^40^, we speculate that a rearrangement of membrane lipids results from the interaction of the natively folded mutant protein with the membrane bilayer. We also observed a slight increase of the absorption around 1454 cm^−1^, partly assigned to hydrocarbon chain CH_3_ groups. As discussed for TTR-WT, and highlighted by the analysis reported at the bottom of Fig. 6a, the spectral changes detected in the 1390–1360 cm^−1^ range were also of interest. In particular, starting from 30 min of incubation we detected an upshift to 1389 cm^−1^ of the 1385 cm^−1^ band, suggesting a reduction of protein glycosylation as well as some modifications of lipid interactions. At 30 min, the intensity of the 1377 cm^−1^ band, mainly assigned to the hydrocarbon chain CH_3_ groups^40^, was also increased. At lower wavenumbers (Fig. 6b, left panel, top spectra), we observed a reduction of glycogen absorptions (see bands at ~1154 cm^−1^, 1080 cm^−1^, 1043 cm^−1^, and 1024 cm^−1^), likely accompanied by a phosphorylation event, as indicated by the simultaneous slight increase of the ~1171 cm^−1^ band and the decrease of the ~1154 cm^−1^ absorption^41^.

Interestingly, as discussed for TTR-WT, cell treatment with early (Fig. 6b, middle spectra) or late (Fig. 6b, bottom spectra) amyloid assemblies of TTR-L55P produced minor effects, as compared to the same variant at pH7.0. However, at variance with cells exposed to the protein at pH7.0, the differences observed in particular in the 1500–1350 cm^−1^ spectral range were more evident at the shorter, than at the longer, incubation time. As shown in the pseudoloading plots reported at the bottom of Fig. 6a and b (left panels), the main contributors to the observed spectral differences were the absorptions in the 1475–1471 cm^−1^ and 1458–1457 cm^−1^ ranges, both assigned mainly to the hydrocarbon chain CH_2_ and CH_3_ groups, and the components in the 1081–1077 cm^−1^ and 1158–1150 cm^−1^ ranges, likely reflecting mainly glycogen as well as glucose absorption.

### FTIR analysis of hydrocarbon chain modifications in HL-1 cells exposed to TTR-L55P

**Figure 7:**
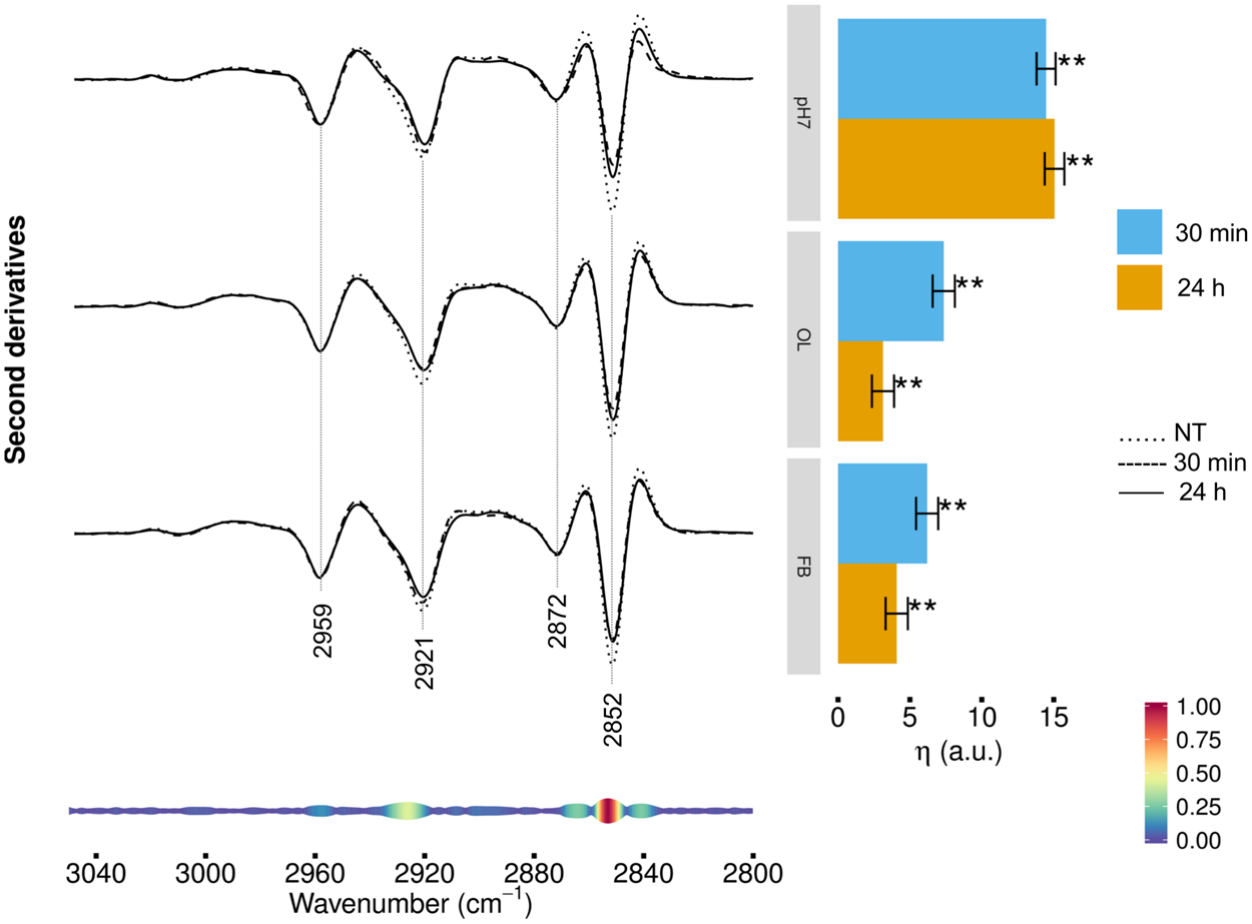
HL-1 cells incubated with TTR-L55P: analysis of the 3050-2800 cm^−1^ CHn stretching range. Left panel: second derivatives of the FTIR absorption spectra of HL-1 cells untreated, incubated for 30 min or 24 h with TTR-L55P in the natively folded (top spectra), oligomeric-like (middle spectra) or fibrillar-like (bottom spectra) conformation. Second derivatives are shown after spectra normalization at the CH_3_ band at 2959 cm^−1^. Representative spectra are shown, computed as average of the three most central spectra within the space formed by the first three PCA-LDA scores. Right panel: differences of the average (across spectra) linear mixed model response variable (η, see Methods for details) between TTR-L55P-treated (pH7, OL, FB at 30 min and 24 h) and control (NT) cells. Error bars show the 95% confidence interval. Stars above the vertical bar indicate the Dunnet’s adjusted two-sided P-values from two-sample Student t-test. P-value: ** < 0.01.

Figure 7 shows the analysis in the 3050–2800 cm^−1^ spectral range, dominated by the absorption of lipid hydrocarbon chains. Cell treatment with natively folded TTR-L55P (pH7.0) (Fig. 7, left panel, top spectra) resulted in a reduction of intensity of the CH_2_ bands^21,25^, particularly that at ~2852 cm^−1^, with a similar extent at the two incubation times. The same spectral changes were found, yet reduced as compared to the protein at pH7.0, in cells incubated with the protein in the oligomeric- (Fig. 7, middle spectra) or fibrillar-like (Fig. 7, bottom spectra) conformation. Moreover, as shown in the graph of Fig. 7 (right panel), the spectral differences were more evident at the shorter incubation time. As highlighted in the pseudoloading plot reported at the bottom of Fig. 7, the main contributor to the observed spectral differences was the CH_2_ absorption around 2852 cm^−1^. This result suggests that cell interaction with the mutant protein results, in particular, in a modification of the length of the lipid hydrocarbon chain, which likely affects membrane fluidity.

## Discussion

In our study, we investigated by FTIR microspectroscopy the effects induced on HL-1, cardiomyocytes, by the treatment for 30 min or 24 h with either TTR-WT and TTR-L55P variant natively folded, or in different, early or late, amyloid aggregated conformations. The effects were traced back to modifications in lipid bilayer fluidity/compactness and in cell metabolic/phosphorylation status. These modifications were correlated with cell viability associated with ROS and free Ca^2+^ levels^11^. FTIR microspectroscopy is a powerful approach to investigate *in situ* the modifications occurring in intact cells exposed to harmless or toxic protein aggregates, as well as to their natively folded monomeric counterparts. It may provide information useful to complement, and to insert into a wider context, the modifications usually detected by commonly used cell biology and microscopy techniques.

For both TTR-WT and TTR-L55P, the major effects were observed when the protein was administered to cells in the natively folded form at pH7.0, while the effects resulting from cell exposure to the protein in the early and late amyloid conformations were milder and in some cases displayed a different timing respect to those elicited by the natively folded protein. Indeed, the harmful effects of the misfolded proteins may be due to the loss of biological function of the protein and/or to a gain of toxic function due to the intrinsic toxicity of amyloid aggregates. The reduction of the cellular functions observed in cells exposed to amyloid aggregates could be a consequence of the loss of the physiological role of TTR in amyloid conformation. However, it is worth noting that, in some cases, minor, yet significant, spectral changes were detected in cells exposed to the amyloid species, indicating that oligomers-like and fibrils-like assemblies can also induce specific responses. In particular, we found that, already after 30 min of incubation, TTR-WT, only when administered in the natively folded form at pH7.0, affected significantly the whole content in secondary structures of cell proteins (see Fig. 2). In particular, it increased the content of α-helix/random coil structures with simultaneous decrease of intramolecular, and increase of intermolecular, β-sheets, a conformation which is typical of protein aggregates and often of protein-protein interactions. Overall, the modifications in protein secondary structures in TTR-exposed cells could be attributed to changes of cell biochemical activities. Indeed, the majority of signal transduction pathways involve protein-protein interactions occurring as a result of intermolecular physical forces and spatial complementation between specialized domains or motifs in the involved proteins. Interactions between the hydrogen-bonding edges of β-strands in different protein chains represent an important mode of protein-protein interaction. These intermolecular β-sheet interactions are frequent in protein quaternary structures^42^, in interactions among different proteins and in protein aggregation. Overall, they play a central role in many biological processes but also in many different diseases, ranging from cancer to amyloid diseases. For these reasons, these interactions are disfavored by protein evolution that worked, among others, to hinder inappropriate interactions among proteins with significant β-sheet content^43^.

The physiological function of TTR is to transport, in the serum and the cerebrospinal fluid, thyroxin and retinol bound to retinol-binding protein (RBP). However, little is known regarding the downstream signaling pathways triggered by wild type TTR in physiological conditions in cardiomyocytes. Recently, it has been reported that TTR can play roles independent of its ligands, spanning from neuroprotection in Alzheimer and schizophrenia to the involvement in memory and learning^44,45^. In the case of cardiac cells, it has been reported that two TTR variants, the amyloidogenic V122I TTR and the non-amyloidogenic T119M TTR variant, interact at the cell membrane of human cardiomyocytes at different sites and with different fates. In fact, the non-amyloidogenic variant is internalized undergoing lysosomal degradation, whereas the amyloidogenic variant initiates the cytotoxic cascade at the cell membrane^46^. These data support the idea that different TTR species interact differently at the cell membrane eliciting different physiological or pathological effects. Finally, TTR binds megalin (LRP-2), RAGE (receptor for advanced glycation end products) and IGF-IR (insulin-like growth factor 1 receptor) and has been described as a new neurotrophic factor able to promote neurite outgrowth in physiological conditions through upregulation of intracellular calcium and the Src/ErK/Akt/CREB pathway in a megalin-dependent manner^47^.

We can also speculate that natively folded TTR-WT is internalized in a subcellular environment that causes protein misfolding and aggregation. The observed protein aggregation might therefore be due to the induced stress to endoplasmic reticulum (ER) that could activate the unfolded protein response^48^. Indeed, similarly to the T119M variant natively folded TTR-WT can also be internalized in a subcellular environment^11^. In chicken oocytes, TTR internalization has been described to occur through a clathrin-dependent pathway mediated by a specific receptor^49^. In pancreatic β-cells TTR binds at the cell surface to Grp78, an ER chaperone found also at the plasma membrane involved in receptor-mediated endocytosis^50^. Moreover, ER Grp78 acts as a protective protein facilitating degradation of harmful, misfolded TTR^50^. ER has an important role in quality control of cell proteins^51^ and in control of Ca^2+^ homeostasis in cardiomyocytes^52^. During cardiac stress (oxidative stress, ischemia, calcium depletion), ER function is disturbed and accumulates unfolded or defective proteins, an ER stress condition that eventually triggers the unfolded protein response (UPR)^53^ and the endoplasmic reticulum associated protein degradation (ERAD) pathway^54^. Although the UPR is considered a compensatory mechanism, its prolonged activation triggers pro-apoptotic signaling and cell death^55^. Recent studies suggest that many cardiovascular diseases, such as cardiac hypertrophy, ischemia, diabetes, and chemotherapy, result in increased ER stress^48,56^.

All these effects may, in some way, contribute to explain the significant modifications we detected in HL-1 cardiomyocytes exposed to natively folded TTR-WT and TTR-L55P and, to a lesser extent, to their misfolded and aggregated derivatives. The cytotoxic effects we described in HL-1 cells were related to their exposure to TTR-WT aggregates and were dependent on the time of exposure, whereas the natively folded protein was harmful. When the cells were treated with the L55P variant, not only the amyloidogenic conformers resulted toxic but also the natively folded mutant at pH 7.0, in agreement with its amyloidogenic propensity. The observed toxicity was related, at least in part, to the increased levels of pro-oxidant metabolites that trigger oxidative stress, together with altered Ca^2+^ homeostasis^11^. We also found increased autophagic vacuoles in cells exposed for 24 h to TTR-L55P at pH 7.0, while no significant changes were observed when the cells were treated with early or late amyloid aggregates with respect to control cells. Previous results from Gonçalves *et al*.^57^ pointed out that enteric glial cells are able to internalize TTR *in vivo* in a FAP mouse model, suggesting that oligomer uptake could impair the autophagic machinery leading to p62 accumulation and eventually to cell death. In our case, the small amounts of oligomers found in the sample of the amyloidogenic variant at pH 7.0 could enhance autophagosome generation to facilitate aggregate removal from the cells. On the contrary, cell treatment with the oligomeric- or fibrillar-like samples by altering Ca^2+^ homeostasis and energy metabolism^11^ could impair the energy-driven autophagic machinery leading to accumulation of aggregates, oxidative stress and apoptotic cell death.

The cell lipid response was also affected after 30 min of cell treatment with both TTR-WT and TTR-L55P in the various forms (see Figs 3a, 4, 6a and 7). Again, major changes were detected in cells treated with the natively folded proteins, even though - at the longer incubation time - significant variations were observed also in cells exposed to amyloid aggregates. In particular, a reduction of hydrocarbon chain length was detected, accompanied by a reorganization of the cholesterol content inside the bilayer, as observed in particular for the WT-TTR (see Fig. 4); this finding agrees with the data showing the key role of cholesterol in TTR interaction at the cell membrane and subsequent internalization^46^.

Recently, lipid-rafts, ordered membrane dynamic functional domains enriched in proteins, sterols and sphingolipids, where key cellular processes are compartmentalized, are emerging as important structural components for cell interaction and transmembrane signaling in response to various stimuli in a variety of cell types^58,59^. These lipid platforms may recruit or aggregate various receptor and signaling molecules such as AMPA and NMDA receptors, trimeric G proteins, small G proteins, sphingomyelin, tyrosine kinases, NADPH oxidase subunits, phosphatases, and many others, resulting in ion flux regulation and the activation of different signaling pathways. Interestingly, recent data indicate that the balance between long-chain ceramides and short-chain ceramides possibly plays a key role in signal transduction and other cellular processes, even though the underlying molecular mechanisms are largely unknown. Long- and short-chain ceramides show entirely different effects on the physical properties of lipid bilayers: long-chain ceramides form ceramides-enriched domains highly ordered and with low dynamics, short-chain ceramides do not form a separate phase but alter the physical properties of the liquid – ordered domains decreasing their stability and viscosity, and perturbing lipid packing^60^. The reduction of hydrocarbon chain length detected in cells exposed to native and aggregated TTR could agree with this scenario (see Figs 4 and 7).

Another very important spectral modification we observed was the reduction of glycogen content and, in general, of carbohydrate absorption, starting from 30 min of cell incubation, even in this case in cells exposed to natively folded TTR (see Figs 3b and 6b). We noticed that the glycogen/carbohydrate decrease matched a parallel increase of phosphorylation, and a “loss” of glycosylation, of cell proteins. The observed prompt reduction of glycogen level should be a consequence of the cell response aimed at restoring the altered Ca^2+^ homeostasis^15^, which needs ATP for active transport. The latter could also explain the lack of the energy needed to support autophagy in the exposed cells. The observed variations of glycogen and, in general, of carbohydrate absorption in exposed HL-1 cells (a cell type particularly enriched in glycogen) could, in effect, result from some variation of protein glycosylation.

Interestingly, an analogous response apparently involving the same cellular processes observed in cells treated with TTR-WT was found, with some important differences, when the cells were exposed to TTR-L55P. Indeed, TTR-L55P, also in the aggregated forms, affected significantly the cell response, yet at lower extent compared to the natively folded protein. In particular, we found that the TTR-L55P amyloids induced a more important lipid response (Fig. 7) respect to the same species grown from the WT protein (Fig. 4). These results imply that the pathogenic, highly amyloidogenic TTR-L55P variant aggregated into amyloid like assemblies with some peculiar structural properties different from TTR-WT that could affect, in particular, the interaction with the lipid membranes. Recent studies on the molecular determinants of TTR misfolding and aggregation have highlighted that single-point amyloidogenic mutations induce destabilization and dissociation of the tetrameric native state into misfolded or partially unfolded dimeric/monomeric species with enhanced propensity for ordered aggregation into amyloid fibrils^61^. Over 100 mutations of TTR are described for human TTR, most of which are amyloidogenic, and amongst these TTR variants, L55P is associated with the most aggressive pathology.

In conclusion, the FTIR approach coupled to multivariate analysis is a powerful technique, complementary to biochemical analysis, to monitor at a different level global structural and chemical modifications in cells exposed to native and variously aggregated polypeptides. This spectroscopic approach is able to provide new insights and clues on the molecular events resulting in functional modifications, impairment and demise of cells experiencing protein/peptides in physiological or pathological conformations.

## Methods

### TTR samples

Recombinant TTR-WT and TTR-L55P were expressed and purified according to Mangione *et al*.^62^. Lyophilized TTR was dissolved at 1.6 mM in 30 mM sodium phosphate buffer, pH 7.0 and its aggregation was primed by adding 100 mM sodium acetate buffer, pH 4.0. TTR-WT amyloid aggregation was performed by incubating the protein at 37 °C and pH 4.0, whereas the TTR-L55P aggregation was performed upon incubation at 37 °C and pH 5.0, as previously reported^63^. After 24 h of aggregation we obtained solutions enriched in oligomer-like amyloid assemblies for both proteins, while enriched fibrillar-like amyloid aggregates of TTR-WT and TTR-L55P were obtained after 72 or 96 h of aggregation, respectively.

### Transmission Electron Microscopy

5.0 μl aliquots of TTR kept under aggregation conditions were withdrawn at different times, loaded onto a formvar/carbon-coated 400 mesh nickel grids (Agar Scientific, Stansted, UK) and negatively stained with 2.0% (w/v) uranyl acetate (Sigma-Aldrich). The grid was air-dried and examined using a JEM 1010 transmission electron microscope (TEM) at 80 kV excitation voltage.

### Dynamic light scattering

Dynamic light scattering (DLS) measurements were performed in a low-volume quartz cuvette (Hellma Analytics, Müllheim, Germany) using a Zetasizer Nano S DLS device from Malvern Instruments (Malvern, Worcestershire, UK) thermostated at 37 °C with a Peltier system. Size distributions by intensity and total light scattering intensity were determined in 10 acquisitions (cell position 4.2 cm, attenuator index 7) of 10 s each, over a period of 10 min. The reported data are the average of three independent measurements.

### FTIR spectroscopy of TTR *in vitro*

The aggregation kinetics of TTR-WT and TTR-L55P were studied by FTIR measurements in attenuated total reflection (ATR) as previously reported^11,64^. In particular, 3 µL of sample aliquots were deposited on the single reflection diamond crystal of the ATR device and the FTIR spectra were collected after solvent evaporation. A 670-IR spectrometer (Varian Australia, Mulgrave, Australia) equipped with a nitrogen-cooled mercury cadmium telluride detector was used under the following conditions: 2.0 cm^−1^ spectral resolution, 25 kHz scan speed, 1000 scan coadditions, and triangular apodization. The second derivatives of the measured spectra were obtained after Savitsky-Golay smoothing using the Resolutions-Pro software (Varian Australia).

### Isolation and culture of HL-1 cardiomyocytes

HL-1 mouse atrial myocytes were obtained from Dr W. C. Claycomb (Louisiana State University Health Science Center, New Orleans, LA, USA) and grown in T25, gelatin/fibronectin-coated flasks, as previously described^65^.

### MTT assay

Cell viability was assessed by the 3-(4,5-dimethylazol-2-yl)-2,5-diphenyl tetrazolium bromide (MTT) assay optimized for the cell line used in the experiments. Briefly, HL-1 cells were seeded into 96-well plates at a density of 6000 cells/well in fresh complete medium and grown for 48 h. Then, the cells were treated for 30 min or 24 h with 10 μM TTR-WT or TTR-L55P at different times of aggregation. After 24 h or 30 min of incubation, the culture medium was removed and the cells were incubated for 1 h at 37 °C in 100 μl of serum-free DMEM without phenol red, containing 0.5 mg/ml MTT. Then, 100 μl of cell lysis solution (20% SDS, 50% N,N-dimethylformamide) were added for 2 h and the absorbance of the blue formazan was read at 570 nm using a spectrophotometric microplate reader. The final absorption values were calculated by averaging each sample in triplicate and subtracting blank average (100 μl of MTT solution + 100 μl of lysis solution).

### Reactive oxygen species measurement

The intracellular levels of ROS were determined using the fluorescent probe 2′,7′-dichlorofluorescin diacetate, acetyl ester (CM-H_2_ DCFDA; Molecular Probes), a cell-permeant indicator for ROS that is not-fluorescent until removal of the acetate groups by intracellular esterases and subsequent oxidation. The latter can be detected by monitoring the increase in fluorescence at 538 nm. The HL-1 cells were plated at a density of 10000 cells per well on 96-well plates. At the end of cell exposure to the aggregates, 10 μM DCFDA in DMEM without phenol red was added and the fluorescence values at 538 nm were read by a Fluoroscan Ascent FL (Thermo-Fisher) reader after 30 min.

### Analysis of autophagic vacuoles

The Cyto-ID® Autophagy Detection Kit (Enzo Life Sciences) was used to monitor autophagy induction by fluorescence microscopy. The cells were plated for 24 h in 24-well plates containing coverslips and then treated with 10 μM natively folded or differently aggregated TTR-WT or TTR-L55P. Then, the medium was removed and the cells were washed twice with 100 μl of PBS and then incubated for 30 min at 37 °C in the dark with 100 μl of freshly diluted Cyto-ID® Green Detection Reagent (1:330). Finally, the cells were washed with Assay Buffer and analyzed by using a confocal Leica TCS SP5 scanning microscope (Mannheim, Germany) equipped with an argon laser source for fluorescence measurements at 488 nm and a Leica Plan Apo 63 × oil immersion objective. A series of optical sections (1024 × 1024 pixels) 1.0 μm in thickness was taken through the cell depth for each examined sample. To quantify the green fluorescent signal, 10–22 cells were analyzed in each experiment using ImageJ software (NIH, Bethesda, MD).

### Statistical analysis

The results of cell viability and antioxidant activities are expressed as mean ± SEM. The statistical analysis of the results was carried out with Graph Pad software and based on Analysis of Variance (ANOVA), one way ANOVA followed by Dunnet’s multiple comparisons test. Statistical significance was set at p < 0.05.

### FTIR microspectroscopy of intact cells

FTIR microspectroscopy was performed on HL-1 cells treated for 30 min or 24 h with TTR-WT and TTR-L55P in the native (pH7), oligomeric- or fibrillar-like conformation. For comparison, we also measured untreated cells. Before measurements, the cells were washed three times in a physiological solution (0.9% NaCl in distilled H_2_O) to eliminate medium contamination. 3 μl of the cell suspension were then deposited onto a BaF_2_ window, and dried at room temperature for at least 30 min to eliminate the excess of water. FTIR absorption spectra were acquired in transmission mode, in the 4000–700 cm^−1^ spectral range, by a Varian 610-IR infrared microscope coupled to the Varian 670-IR FTIR spectrometer (both from Varian Australia Pty Ltd), equipped with a mercury cadmium telluride nitrogen-cooled detector. The variable microscope aperture was adjusted to ~100 μm × 100 μm. Measurements were performed at 2.0 cm^−1^ spectral resolution; 25 KHz scan speed, triangular apodization, and by the accumulation of 512 scan co-additions. Moreover, the spectra affected by Mie scattering were corrected using the algorithm developed by Bassan and colleagues^66^ and the spectral range of the correction - originally in the 4000–1000 cm^−1^ range- was extended in our laboratory down to 800 cm^−1^. Then, the corrected spectra were normalized at the Amide I band area, for comparison and the second derivative analysis was performed (after a 13-point smoothing of the measured spectra) by the Savitzky-Golay method (3rd polynomial, 9 smoothing points), using the GRAMS/32 software (Galactic Ind. Corp., Salem, NH, USA). For each sample, we repeated several measurements by selecting different areas on the same sample through the variable diaphragm aperture of the infrared microscope. Furthermore, to evaluate the reproducibility of the results, we performed three independent experiments.

### Multivariate analysis of the FTIR data

FTIR raw spectra were first auto-scaled (zero mean, and standard deviation one) and then subjected to principal component analysis (PCA). Then, the linear discriminant analysis was applied to the spectra projected on the low dimensional principal components (PCs) space. In this case, LDA could not be applied directly to the raw spectra, since the number of observations (the spectra) was smaller than the number of variables (the discretized wavenumbers). LDA was iteratively applied using an increasing number of PCs (from 3 to 20) and the first K principal components maximizing the LDA classification accuracy were retained. K may be different for each independently analyzed spectral range. Classification accuracy was computed as the number of correctly assigned cases over the total number of cases. Representative spectra were selected as the average of the three most central spectra in the score space formed by the first three PCA-LDA score components. Coefficients for the original predictors have been estimated as $coef{f}_{n,Nlda}={W}_{n,K}\times {\beta }_{K,Nlda}$, where *W*_*n,K*_ are the first K eigenvectors retained and *β*_*K*_,_*nlda*_ are the LDA coefficients^8^. Pseudoloadings were computed by averaging across coefficients as $P{L}_{n}={\sum }_{1}^{Nlda}coef{f}_{l}\sqrt{{\lambda }_{l}}$, being *λ*_*l*_ the eigenvalues associated to the l-th discriminant component.

The first LDA score was used to build a linear mixed model (LMM)^9^. LMMs allow studying relationships between a response variable and some covariates taking into account for the variability among subjects, replicas or other units. LMM incorporates both the so called fixed-effects and random-effects. Fixed-effects parameters model categorical variable whose set of possible levels is fixed and reproducible (*e.g*. treatments). If, for a given categorical covariate, the observed levels represent a random sample from the set of all possible levels, we incorporate random-effects in the model (*e.g*. subjects)^67^. In our case, the fixed-effects are: Group = (NT, FB, OL, pH7), Time = (30 min, 24 h), Measurements = (M1, M2, …, Mmax). The measurements were repeated on at least three cell samples obtained from different cell culture replicas. The variability within the different cells samples can be modeled by the random-effect: Cell = (1, 2, 3, 4, …N). As response variable the first PCA-LDA score (*y*_*ijkl*_) was used. The final model can be written as:

$$\begin{array}{rcl}{y}_{ijkl} & = & u+{\beta }_{G}Grou{p}_{i}+{\beta }_{T}Tim{e}_{j}+{\beta }_{M}Mea{s}_{k}+{\beta }_{GT}(Grou{p}_{i}Tim{e}_{j})\\  &  & +\,{\beta }_{GM}(Grou{p}_{i}\,Mea{s}_{k})+{\beta }_{TM}(Tim{e}_{j}\,Mea{s}_{k})\\  &  & +\,{\beta }_{GTM}(Grou{p}_{i}\,Tim{e}_{j}\,Mea{s}_{k})+{\gamma }_{0l}+{\gamma }_{1jl}+{\gamma }_{2kl}+{\varepsilon }_{ijkl}\end{array}$$

A similar model can be obtained using a three-ways repeated measurements ANOVA with one between-subject factor (Group) and two within-subjects factors (Time, Measurements)^68^. The main advantage of LMM over ANOVA is the possibility to easily treat unbalanced data sets^68^. Least-square means have been estimated on the LMM response variable for each group and time combination^69^. Then, the least-square means of the treated cases (FB, OL, pH7) were compared to the control case (NT) as ${\eta }_{G^{\prime} ,T}=LS{M}_{G^{\prime} T}-LS{M}_{NT,T}$, with G′ = (FB, OL, pH7), T = (30 min, 24 h), LSM = least-square mean. The statistical significance of this difference was assessed using a two-sample paired t-tests. The P-value was computed using Dunnet’s adjustment^70^. η_G′,T_ is a measure of the diversity between the treated and the control cases on the first discriminant function. The larger is η_G′,T_, the more different are the spectra measured on the cells treated with FB, OL or the protein at pH7.0 compared to the untreated cells. Wavenumbers whose associated pseudoloading was greater than 0.6 have been considered as the most relevant ones. This threshold was chosen as for all analysed cases, the third quartile of the pseudoloadings distribution was approximately 0.6.

## Electronic supplementary material

Supplementary materials
