# Supplementary material for: A FTIR microspectroscopy study of the structural and biochemical perturbations induced by natively folded and aggregated transthyretin in HL-1 cardiomyocytes
Source: Sci Rep. 2018 Aug 21;8:12508. doi: 10.1038/s41598-018-30995-5 (PMC6104026; doi:10.1038/s41598-018-30995-5)
Supplement: Supplementary file 1 — Supplementary materials [file 41598_2018_30995_MOESM1_ESM.pdf]

SUPPLEMENTAL MATERIALS

FOR:

A FTIR microspectroscopy study of the structural and biochemical perturbations induced by natively folded and aggregated transthyretin in HL-1 cardiomyocytes.

Diletta Ami[1]*, Paolo Mereghetti[1], Manuela Leri[2, 3], Sofia Giorgetti[4], Antonino Natalello[1], Silvia Maria Doglia[1], Massimo Stefani[3, 5], Monica Bucciantini[3, 5]*

[1] Department of Biotechnology and Biosciences, University of Milano-Bicocca, Piazza della Scienza 2, 20126, Milano, Italy

[2] Department of Neuroscience, Psychology, Area of Medicine and Health of the Child of the University of Florence, Viale Pieraccini, 6 - 50139 Florence, Italy

[3] Department of Experimental and Clinical Biomedical Sciences, University of Florence, Viale Morgagni 50 - 50134 Florence, Italy

[4] Department of Molecular Medicine, Unit of Biochemistry, University of Pavia, Viale Taramelli 3/B 27100 Pavia, Italy

[5] Interuniversity Center for the Study of Neurodegenerative Diseases (CIMN), Florence, Italy

*corresponding Authors

Diletta Ami, Ph.D.
University of Milano-Bicocca
Department of Biotechnology and Biosciences
Piazza della Scienza 2, 20126 Milano
Phone +39 02 64 48 34 61
diletta.ami@unimib.it

Monica Bucciantini
Prof. Monica Bucciantini, Ph.D.
University of Florence
Department of Experimental and Clinical Biomedical Sciences
Viale Morgagni, 50
50134 Florence, Italy
Phone+39-055-2751250
monica.bucciantini@unifi.it

Figure S1

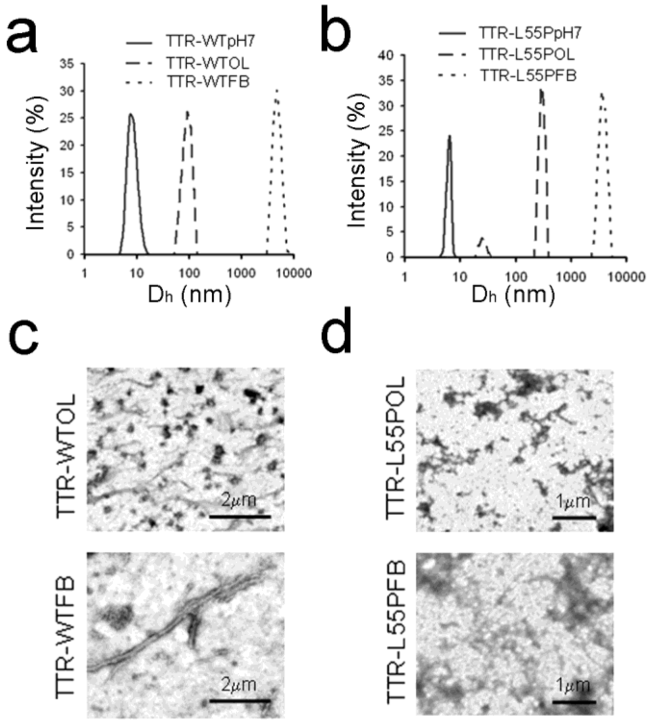

Figure S1. Characterization of TTR-WT and TTR-L55P variant in different conformational states by Dynamic light scattering (DLS) and Transmission electron microscopy (TEM). DLS of TTR-WT (a) and of TTR-L55P (b) in native (pH7), oligomeric- (OL) and fibrillar-like (FB) conformation. TEM of TTR-WT (c) and TTR-L55P (d) in oligomeric- (OL) and fibrillar-like (FB) conformation. Scale bar is shown in each micrograph.

Figure S2

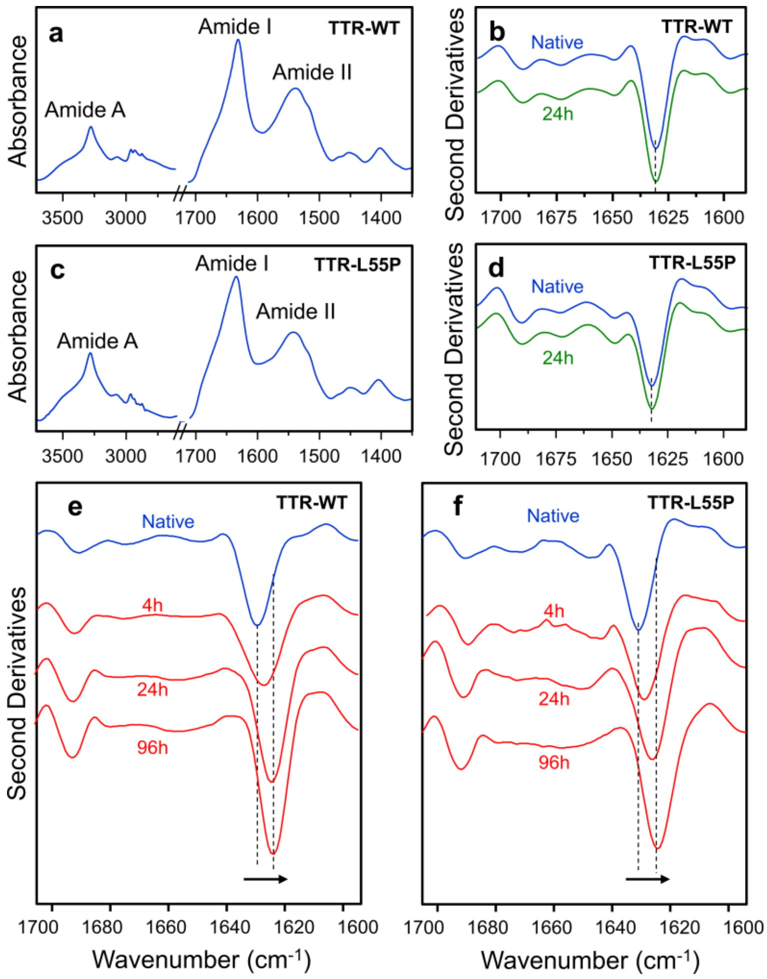

Figure S2. Characterization of TTR-WT and TTR-L55P variant in different conformational states by ATR-FTIR spectroscopy. (a) ATR-FTIR absorption spectrum of native TTR-WT (blue spectrum). (b) Second derivatives of the absorption spectra of native TTR-WT measured before (blue spectrum). and after incubation for 24 hours at neutral pH and at 37°C (green spectrum). The dashed vertical line indicates the Amide I component assigned to the native β-sheet structure, as previously reported[11]. Spectra were reported in the Amide I region. No detectable spectral changes were observed after 24 hours incubation, indicating protein stability under our experimental conditions. (c, d) Absorption (c) and second derivative (d) spectra of TTR-L55P variant showed as in (a) and (b), respectively. Second derivative spectra of TTR-WT (e) and TTR-L55P variant (f) reported at different times of incubation at acidic conditions (red spectra). The spectra of the native proteins (blue spectra) are also showed for comparison. During incubation, the downshift of the component assigned to native β-sheets (see arrows) indicates a rearrangement of the β-sheet structures in the growing aggregates.

Figure S3

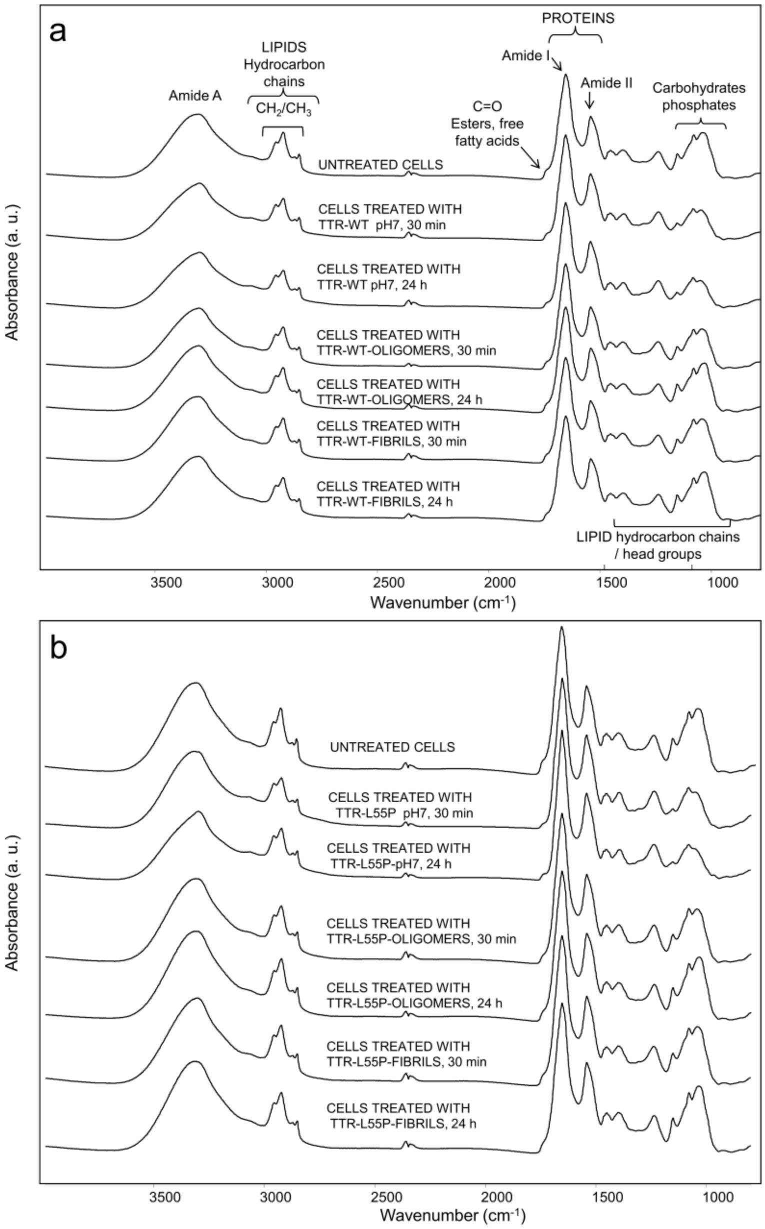

Figure S3. Representative FTIR absorption spectra of HL-1 intact cells treated with TTR-WT (a) and TTR-L55P (b), in the different conformational states, at the indicate times of incubation. The spectra have been corrected for the Mie scattering[66]. In (a) the assignment of the bands due to the main biomolecules discussed in the text has been reported.

Figure S4

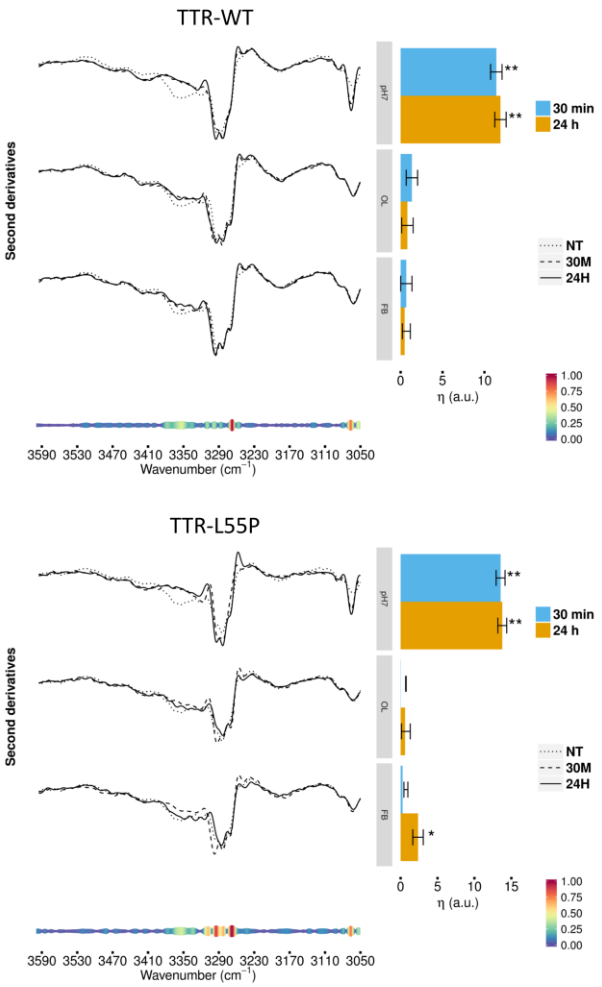

Figure S4. FTIR Amide A band analysis. Left panel: second derivatives of the FTIR absorption spectra of HL-1 cells untreated (dotted line), incubated for 30 min (dashed line) or 24 h (continuous line) with TTR-WT and TTR-L55P, in native conformation (top spectra), in the early oligomeric (middle spectra) or in the late fibrillar-like (bottom spectra) form. Second derivatives are shown after spectra normalization at Amide I band area. Representative spectra are shown, computed as average of the three most central spectra within the space formed by the first three PCA-LDA scores. At the bottom, the pseudoloadings are shown as coloured bands: larger, red-coloured bands indicate important wavenumbers, while smaller, blue-coloured bands indicate unimportant wavenumbers (see bottom-right colour bar). Right panel: differences of the average (across spectra) linear mixed model response variable (η, see Methods for details) between TTR treated (pH7, OL, FB at 30 min and 24 h) and control (NT) cells. Error bars show the 95% confidence interval. Stars above the vertical bar indicate the Dunnet's adjusted two-sided P-values from two-sample Student t-test. P-value: ** < 0.01.
